# Safety and efficacy of the HilumDirect uVATS for small cell lung cancer: a retrospective study

**DOI:** 10.3389/fsurg.2025.1666087

**Published:** 2025-12-18

**Authors:** Xining Zhang, JingWei Liu, Shijie Zhang, Jian Li

**Affiliations:** Department of Thoracic Surgery, Peking University First Hospital, Beijing, China

**Keywords:** small cell lung cancer (SCLC), surgery, uVATS, multidisciplinary approach, surgical techinque

## Abstract

**Background:**

Despite advancements in multidisciplinary treatments, long-term survival rates for small cell lung cancer (SCLC) patients remain poor, and the role of surgical resection has been debated since the late 20th century. A more comprehensive surgical approach is necessary to enhance local control and improve outcomes.

**Method:**

The records of the patients who received surgical treatment between January 2015 and December 2020 were collected and analyzed with propensity-score matching. The preoperative characteristics, short-term outcomes, and long-term survival were analyzed and compared between the HilumDirect uVATS and the conventional uVATS groups.

**Results:**

Preoperative variables were well-balanced between the two groups. The HilumDirect uVATS group showed a numerically higher lymph node yield. Patients in the HilumDirect uVATS group pointed toward a numerical difference in cancer-specific survival (CSS) and recurrence-free survival (RFS) compared to the Conventional resection group (CSS, 50.82% vs. 44.09%, *p* = 0.890, RFS, 42.86% vs. 38.46%, *p* = 0.904). This numerical difference was consistent in both lymph node-negative (CSS: 72.70% vs. 51.60%, *p* = 0.287, RFS: 58.30% vs. 47.60%, *p* = 0.654) and node-positive subgroups (CSS: 35.70% vs. 33.30%, *p* = 0.869, RFS: 31.20% vs. 22.22%, *p* = 0.833). Competing risks analysis suggested a non-significant difference in the lower risk of distal metastasis of the HilumDirect uVATS (Subdistribution hazard ratio of Conventional uVATS: 2.164, 95% CI 0.741–6.318, *p* = 0.158).

**Conclusion:**

The HilumDirect uVATS resection can be safely performed by experienced surgeons with minimal invasiveness. Our findings suggest that selected SCLC patients, particularly those with positive lymph nodes, may benefit from this approach. However, further studies are warranted to confirm these results.

## Background

Globally, there are approximately 2.21 million cases of lung cancer diagnoses and 1.80 million deaths (1). Small cell lung cancer (SCLC) accounts for about 10%–15% of primary lung malignancies, characterized by rapid growth and early metastasis (2). Current guidelines recommend concurrent chemoradiotherapy (CRT) for node-positive SCLC (3–5), which has yielded 5-year survival rates of 16%–26% (6). Surgical resection, combined with CRT as part of a multidisciplinary treatment approach, is only recommended in clinical stages I and II disease (cT1-T2N0), as none of the existing large randomized controlled trials (RCT) (7, 8) or the recent Cochrane systematic review (9) demonstrated a survival benefit from surgical resection for SCLC.

In contrast, retrospective studies have suggested acceptable outcomes in surgery for early-stage disease (10–13). However, despite the barely acceptable results, the recurrence rate of surgically treated SCLC remains high. According to a retrospective study by the Mayo Clinic, the postoperative recurrence rate of the combined treatment of surgery and CRT was reported to be as high as 50% at 3 years (median RFS, 2.4 years) (14). This absence of a synergistic effect of surgery and CRT in SCLC treatment, which has also been suggested by previous RCTs (7, 8), could be at least partially due to the method of surgical resection. For instance, inappropriate surgical manipulation can theoretically dislodge tumor cells into the bloodstream and lymphatic system (15); this effect might be more significant in highly aggressive diseases, such as SCLC, thus leading to postoperative distant metastasis.

Given the limitations of contemporary SCLC resection methods and the high postoperative recurrence rates, there is an urgent need for innovative surgical techniques that ensure precise, complete resection while minimizing the risk of circulating tumor cell dissemination. Although recent advancements and a deepening understanding of VATS have shown promising results in the radical resection of SCLC, controversies still exist regarding the effectiveness of this procedure. These include limited access to mediastinal lymph nodes (16), challenges with instrument maneuverability (17), and concerns about intraoperative tumor handling (18). Given the aggressive nature of SCLC, a more refined surgical approach that enhances oncologic resection while maintaining minimal invasiveness is necessary. HilumDirect uVATS may provide advantages for SCLC management by enabling a single small-sized incision with direct access to the lung hilum. This approach facilitates improved precision, potentially reducing the risk of tumor cell dissemination and enabling more thorough lymph node clearance. By minimizing tumor manipulation, this technique could enhance local control outcomes, lower recurrence rates, and offer a promising alternative to conventional resection methods for SCLC. By comparing outcomes of HilumDirect uVATS with conventional uVATS, this study seeks to provide insights into optimizing surgical intervention for SCLC within a multimodal treatment framework.

## Materials and methods

### Patient selection

The records of patients who underwent curative resection for SCLC between January 1, 2015, and December 31, 2020, at our center were collected and analyzed. Our institutional ethics committee reviewed and approved this study (approval no. 2025-070), and informed consent was obtained from each patient. The inclusion criteria were (1) pulmonary SCLC confirmed by the postoperative pathological study, (2) age 18 years or older, (3) no distal metastasis preoperatively, and (4) curative intent resection conducted. The exclusion criteria were (1) no SCLC component found in the postoperative resection, (2) death within 1 month after surgery, and (3) failure to achieve gross complete resection according to the surgical records.

### Staging work-up and postoperative care

The perioperative evaluation included the ECOG (Eastern Cooperative Oncology Group) performance status scale, electrocardiogram, lung function tests, arterial blood gas analysis, echocardiogram, CT (computed tomography) scan of the chest and upper abdomen, cranial magnetic resonance imaging, and a radioisotope bone scan. Whole-body Positron-emission-tomography computed tomography (PET-CT), bronchoscopy biopsy, and Endobronchial Ultrasound-guided Transbronchial Needle Aspiration (EBUS-TBNA) were routinely recommended. The staging workup was then performed according to the AJCC's eighth edition TNM staging system. All treatment decisions were made by the multidisciplinary board, which consisted of representatives from thoracic surgery, oncology, radiology, and radiotherapy. Surgical treatment was recommended for patients diagnosed with SCLC, along with neoadjuvant chemotherapy and adjuvant chemoradiotherapy.

If needed, the patient would receive postoperative treatment, typically on the first to third postoperative day, in the intensive care unit (ICU) or return directly to our ward. Routine postoperative treatments include prophylactic antibiotic administration, intravenous patient-controlled anesthesia, and anticoagulation treatments. The blood routine and biochemistry tests were conducted every other day. The thoracic tube would be removed if (1) the water-sealed level stays still while coughing or speaking, (2) the volume of the drainage fluid is less than 200 mL 24 h prior to the removal, and (3) the drainage fluid is yellow colored. The patient was discharged the day after the thoracic tube removal if no discomfort was reported.

### Method of operation

All HilumDirect uVATS procedures were performed by a single thoracic surgeon who developed the technique and had independently completed over 1,000 lung resections. The conventional uVATS procedures were carried out by six other surgeons with comparable operative volume and technical proficiency.

**Figure 1:**
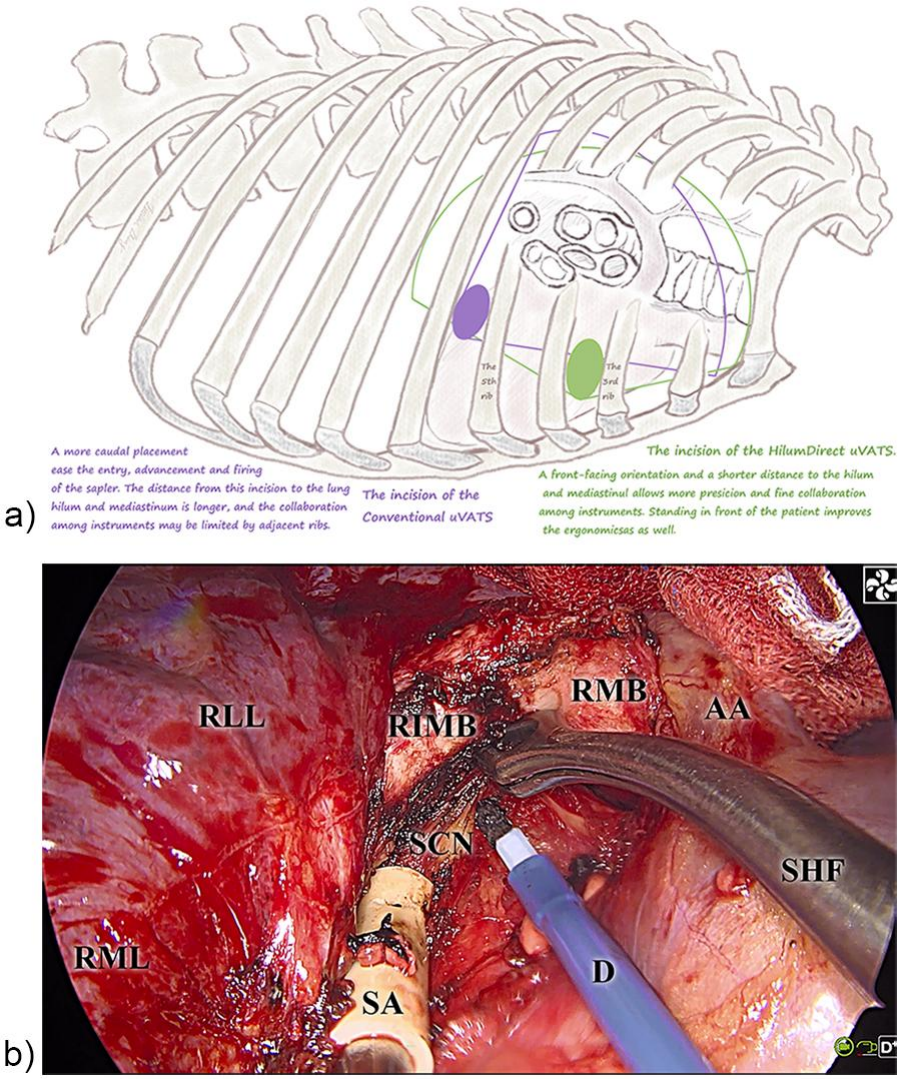
The illustration of the incision of HilumDirect uVATS and conventional uVATS. The drawing and photograph depict the view of the HilumDirect uVATS. **(a)** Illustrates the geographic concept of the HilumDirect uVATS through a drawing. The green oblong along the anterior third intercostal space represents the incision for the HilumDirect uVATS The green fan-shaped area signifies the two-dimensional projection of the operative cone of the HilumDirect uVATS In contrast, the purple oblong and fan-shaped area represent the incision and the two-dimensional projection of the operative cone of the Conventional uVATS Note that from the surgeon's perspective, the incision for the HilumDirect uVATS is straighter, facilitating more complex maneuvers. Additionally, the distance between the incision and the operative field is optimized, enhancing precision. **(b)** Features a photograph capturing the intraoperative view during a HilumDirect uVATS right upper lobectomy, showing a suction tip, a diathermy pen, and thoracoscopic snakehead forceps. This photo highlights the close collaboration of multiple instruments for subcarinal lymphadenectomy through the anterior approach. AA denotes the azygos arch. D refers to the diathermy pen. RIMB indicates the right intermediate bronchus. RMB stands for the right main bronchus. RLL represents the right lower lobe. RML denotes the right middle lobe. SA refers to the suction apparatus tips. SCN indicates the subcarinal lymph nodes. SHF stands for the snakehead forceps.

**Figure 2:**
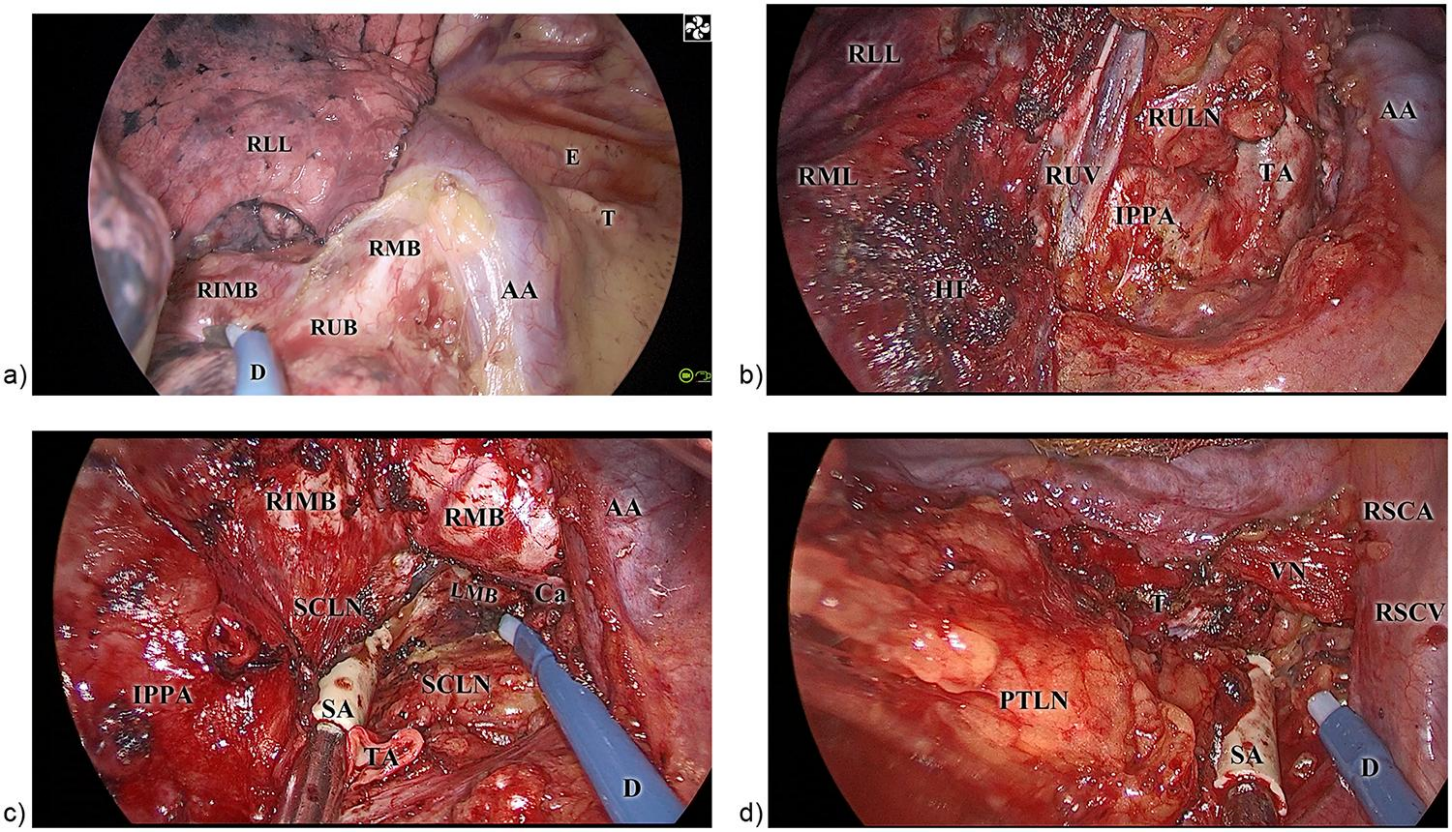
The intraoperative images of the HilumDirect uVATS. The intraoperative images of the HilumDirect uVATS are presented to illustrate the operation method. **(a)** This view shows the right upper posterior lung hilum, which can be easily exposed using the HilumDirect uVATS. Additionally, maneuvers such as dissection, transection, and hemostasis can be performed with precision. **(b)** An intraoperative image of the right upper anterior lung hilum. The main structures of the right upper lobe are optimally exposed, facilitating subsequent transections with ligations or staplers. It is noteworthy that the right upper lobar lymph nodes are managed *en bloc* with the lobe. **(c)** The HilumDirect approach for station 7 lymphadenectomy. The lymph nodes and adjacent connective tissue can be effectively exposed and resected through this approach. Close collaboration among instruments allows for precise margin control and other complex maneuvers. **(d)** The lymphadenectomy view for stations 2R and 4R. This intraoperative photograph emphasizes the optimized exposure of station 2R lymph nodes, extending up to the vagus nerve and the right subclavicular artery, which are typically difficult to access in conventional uVATS. AA, azygos arch; D, diathermy pen; Ca, carina; E, esophagus; HF, the horizontal fissure of the lung; IPPA, the intraparenchymal pulmonary artery; PTLN, the right paratracheal lymph nodes (stations 2R and 4R); RIMB, the right intermediate bronchus; RMB, the right main bronchus; RML, the right middle lobe; RLL, the right lower lobe; RSCA, the right subclavicular artery; RSCV, the right subclavicular vein; RUB, the right upper bronchus; RULN, the right upper lobar lymph nodes (station 12); RUV, the right upper lobar vein; SCLN, the subcarinal lymph nodes (station 7); T, trachea; TA, truncus anterior artery; VN, the right vagus nerve.

The surgery was performed using either the HilumDirect uVATS method or conventional uVATS surgery. In the HilumDirect uVATS group, the procedure was conducted through a 2.–3.5-centimeter incision, parallel to the upper part of the lung hilum, typically at the third or, in rare cases, the fourth intercostal space along the anterior axillary line, where the lateral margin of the pectoralis major is located (Figure 1a). This anterior section of the intercostal spaces was not covered by the serratus anterior muscle. Thus, in these positions, the risk of nerve injury, such as the lateral cutaneous branches of the intercostal nerve, the long thoracic nerve, and other nerves closely associated with the serratus anterior muscle, would be reduced. The prototype of HilumDirect uVATS has been previously introduced and illustrated (19, 20). Beyond the theoretical nerve-protective effect, this approach offers a shorter distance to the lung hilum, main bronchus, and trachea, thereby facilitating the resection of significant parenchyma and lymph nodes. A more anteriorly placed incision also enhances the angle between the chest wall and instruments, reducing the compression of surgical instruments against the ribs and improving the coordination among the instruments (Figure 1b). Given the optimized distance from the incision to the operative field and the larger movement angle of instruments, surgical maneuvers in HilumDirect uVATS can be performed from virtually any direction. Furthermore, the straighter operative axis supports complex surgical movements, including cutting, suturing, ligating, and transfixing, with minimal tissue traction. These ergonomic improvements are especially beneficial for precise diathermy cutting and coagulation, optimizing resection margins while ensuring oncological completeness. For instance, in a right upper lobectomy, upon entering the thoracic cavity and ruling out pleural lesions, the right upper hilum is readily accessible, allowing for diathermy division of the right upper bronchus (Figure 2a). With the bronchus acting as the primary tethering structure, its division enhances the visibility of the pulmonary artery, vein, and underdeveloped fissures (Figure 2b). These structures are then controlled and divided in a systematic manner.

The lymph node dissection in HilumDirect uVATS significantly benefits from the reduced distance and increased instrument mobility. Subcarinal lymph nodes (Station 7) can be approached from both anterior and posterior angles, facilitating complete dissection of subcarinal and adjacent main bronchial nodes (Figure 2c). Upper mediastinal lymph node clearance is notably improved due to the incision's proximity to the trachea, superior vena cava, and azygos arch. This allows for en-bloc dissection of stations 2R, 4R, and upper hilar lymph nodes (Figure 2d). The 3a station and lymph nodes adjacent to the recurrent laryngeal nerve can also be harvested en-bloc if necessary. Post-resection procedures, including stapling of remaining lung tissue and hemostasis, remain consistent with conventional techniques. A comprehensive lobe-specific and lymph node station-specific operative approach is detailed in the Supplementary Material.

In the conventional group, the procedure was performed according to the current expert consensus on uVATS. The incision, typically 3–5 cm long, was made in the fourth or fifth intercostal space along the middle or posterior axillary line. The pulmonary vessels were sequentially isolated, dissected, and ligated using energy devices and staplers. After achieving vascular control, the bronchus was mobilized and stapled. However, the limited angle of entry occasionally posed ergonomic challenges during dissection. Lymphadenectomy was performed in accordance with the current guidelines. For the resection of upper mediastinal lymph nodes, additional retraction or angled instruments were often necessary.

### Follow-up and outcome

Chemotherapy, with or without concurrent radiotherapy depending on the presence of residual disease in the thorax as indicated by the pathological report, was routinely recommended to patients. After discharge, patients would receive follow-up appointments every 3 months during the first 2 years, then every 6 months from the third to the fifth years. Chest CT, abdominal ultrasonography, superficial lymph node imaging, head MRI, and serum tumor marker tests were conducted at each follow-up. The primary outcomes of our study are CSS and RFS. The CSS rate was calculated from the date of surgery to the date of death due to SCLC recurrence, while the RFS rate was determined from the date of surgery to the date of disease recurrence. Patients who remained event-free at the last available follow-up date were right-censored at that date in the survival analysis.

### Statistical analysis

PSM analysis, a method that could limit bias caused by an existing dataset for nonrandom assignment analysis, was utilized to reduce the inherent bias in the retrospective study (21). Propensity scores were calculated using logistic regression based solely on the preoperative characteristics according to the methodology of PSM (22–24), including age, sex, smoking history, preoperative lung function test results, lung cancer-related serum tumor marker antigen levels, neoadjuvant therapy, and tumor diameter. The dataset was complete for all variables used in the propensity score model; therefore, no missing-data handling was required. A 1:1 matched cohort was created by matching patients from the two groups with a caliper width equal to 20% of the standard deviation of the propensity scores, without replacement. The post-matching balance was assessed using the Student's *t*-test or Wilcoxon rank-sum test for continuous variables and the chi-square test or Fisher's exact test for categorical variables.

Competing risks analysis (CRA) was employed to accurately evaluate survival outcomes while accounting for the presence of alternative events that could interfere with or mask the primary endpoint (25). We utilized the Fine and Gray subdistribution hazard model to estimate the cumulative incidence function (CIF) of recurrence types and to compare the subdistribution hazard across groups. The cmprsk package (version 2.2-11) in R was used to perform all competing risks analyses. Gray's test was applied to compare cumulative incidence functions between surgical groups. In this study, only the first event per patient was considered. Events were categorized into three mutually exclusive types: regional recurrence, distal recurrence, and disseminated (regional and distal) recurrence. Those without any event at the last follow-up were treated as censored. Subdistribution hazard ratios (sHRs) were calculated to assess the relative risk of recurrence types between surgical groups.

The CSS and RFS of patients who underwent HilumDirect uVATS and conventional uVATS radical resection for SCLC were calculated and compared using the Kaplan–Meier method in the overall study population and the PS-matched cohort. Covariates selected for multivariable Cox regression were screened through univariable testing at *p* < 0.1. The normality of continuous variables was assessed using the Shapiro–Wilk test. Student's *t*-test and Wilcoxon rank-sum test were utilized to compare normally and skewed distributed continuous variables between groups, respectively. The chi-square test or Fisher's exact test was conducted for categorical variables. For all analyses, a *p*-value <0.05 in a two-tailed test was deemed statistically significant. All analyses were performed using STATA/MP 15.1 software (StataCorp LLC, College Station, TX, USA), the R Project for Statistical Computing (26), and R Studio.

## Results

### Patients' characteristics

A total of 87 SCLC patients who underwent either HilumDirect uVATS or conventional uVATS surgery were identified, including 29 patients who received HilumDirect uVATS and 58 patients who underwent conventional uVATS. The patients who received HilumDirect uVATS had higher levels of Neuron-Specific Enolase (NSE) (15.32 [12.67–20.2] vs. 12.91 [11.46–16.03], *p* = 0.035) and Pro-Gastrin-Releasing-Peptide (Pro-GRP) (118.7 [50.53–272.9] vs. 62.95 [42.6–121.65], *p* = 0.034) (Table 1). Other preoperative variables between the two groups showed no significant difference. PSM generated 28 pairs of patients with well-balanced preoperative characteristics (Table 1).

**Table 1:** Baseline characteristics of patients before and after the propensity score matching.

Variables	Unmatched cohort			Matched cohort		
	HilumDirect uVATS (29)	Conventional uVATS (58)	*p*-value	HilumDirect uVATS (28)	Conventional uVATS (28)	*p*-value
Sex			0.409			0.342
Male	23	50		23	20	
Female	6	8		5	8	
Age (year)	63.07 ± 12.10	62.14 ± 9.08	0.689	62.93 ± 12.30	63.18 ± 9.68	0.932
Smoking (pack-year)	0.94 (0–40)	2.75 (0.69–36.25)	0.474	0.72 (0–40)	2.75 (0–30)	0.820
ECOG			1.000			1.000
0	28	55		27	28	
1	1	3		1	0	
FEV1%	92.6 (77.35–112.15)	91.6 (78.68–104.6)	0.804	90 (77.01–113.23)	93.25 (77.8–109.28)	0.825
DLCO%	81.13 ± 18.20	81.48 ± 16.89	0.930	81.09 ± 18.54	78.97 ± 15.44	0.644
Resection			0.637			0.836
Sublobar resection	1	6		1	1	
Lobectomy	21	37		20	16	
Bilobectomy^a^	6	14		6	10	
Right pneumonectomy	1	1		1	1	
Clinical N2 metastasis			0.871			0.771
No	20	39		19	20	
Yes	9	19		9	8	
Neoadjuvant therapy			0.874			1.000
0	19	37		18	18	
1	10	21		10	10	
CEA (ng/mL)	3.46 (1.99–5.56)	3.27 (2.46–5.4)	0.583	3.25 (19.8–5.33)	3.22 (2.58–5.42)	0.517
CA19-9 (IU/mL)	14.6 (7.92–19.19)	12.7 (7.84–19.21)	0.685	14.47 (7.81–19.28)	11.86 (6.78–25.84)	0.737
SCC (ng/mL)	0.7 (0.5–1.05)	0.8 (0.6–1.2)	0.089	0.7 (0.5–1.08)	0.8 (0.7–1.18)	0.058
Cyfra21-1 (ng/mL)	2.99 (2.09–3.54)	3.07 (2.10–3.82)	0.746	2.95 (2.07–3.57)	3 (1.84–4.23)	0.694
NSE (ng/mL)	15.32 (12.67–20.2)	12.91 (11.46–16.03)	0.035	15.20 (12.67–19.79)	13.04 (11.56–18.55)	0.291
ProPRG (pg/mL)	118.7 (50.53–272.9)	62.95 (42.6–121.65)	0.034	118.5 (49.04–222.6)	62.95 (42.42–180.43)	0.210
Adjuvant chemotherapy			0.402			0.771
No	10	15		9	8	
Yes	19	43		19	20	
Adjuvant radiotherapy			0.409			0.737
No	23	50		22	23	
Yes	6	8		6	5	

ECOG, eastern cooperative oncology group performance score; FEV1%, percentage of predicted 1-second forced expiration volume value; DLCO%, percentage of predicted diffusing capacity of the lungs for carbon monoxide; CEA, carcinoembryonic antigen; CA19-9, cancer antigen 19-9; SCC, squamous cell carcinoma antigen; Cyfra21-1, cytokeratin 19 fragment 21-1; NSE, neuron-specific enolase; PRG, pro-gastrin-releasing peptide.

^a^ The left pneumonectomy was counted within.

### Perioperative outcomes

Table 2 presents the perioperative outcomes of the overall and PS-matched cohorts. While the HilumDirect group has a significantly higher percentage of patients undergoing complex surgery, there is no notable difference in intra-operative parameters (i.e., operative length, bleeding, etc.) and postoperative complication rates between the two groups. The HilumDirect group's ICU stay rate is higher than that of the Conventional group (28% vs. 6%, *p* = 0.037), although this significance did not persist after PSM. There is a numeric difference indicating a greater lymph node yield in the HilumDirect group, although it is not significant (19 [14–25.5] vs. 16.5 [11.75–22], *p* = 0.128). This numeric difference remained consistent in the station-specific sub-variables (N1 lymph node yield, 9 [5.5–10.5] vs. 7 [4–9.5], *p* = 0.183, N2 lymph node yield, 10 [7–16.5] vs. 8.5 [5–14], *p* = 0.254). These numeric differences persisted after PSM.

**Table 2:** Operative parameters before and after the propensity score matching.

Variables	Unmatched			Matched		
	HilumDirect uVATS (29)	Conventional uVATS (58)	*p*-value	HilumDirect uVATS (28)	Conventional uVATS (28)	*p*-value
Complex operation^a^			0.153			0.193
No	24	54		23	27	
Yes	5	4		5	1	
Operative time (minutes)	239 (197.5–274.5)	230 (185–270.75)	0.549	241 (197.25–275.25)	228.5 (183–281)	0.676
Operative bleeding volume (mL)	100 (35–200)	100 (50–162.5)	0.732	100 (27.5–200)	100 (27.5–187.5)	1.000
Operative infusion volume (mL)	1,600 (1,450–1,950)	1,600 (1,375–2,100)	0.872	1,600 (1,425–2,025)	1,600 (1,400–2,000)	0.987
Operative urine output (mL)	400 (275–625)	400 (200–600)	0.950	400 (262.5–550)	425 (262.5–600)	0.459
Chest tube duration (days)	6 (4–7)	4.5 (3–6.25)	0.055	6 (4–7)	4 (3–6.75)	0.062
Postoperative complications			0.328			0.611
No	26	56		25	27	
Yes	3	2		3	1	
ICU Stay			0.037			0.143
No	22	54		21	26	
Yes	7	4		7	2	
Lymphadenectomy N2 stations	4 (3–4)	3 (3–4)	0.340	4 (3–4)	3.5 (3–4)	0.614
Lymphadenectomy N1 stations	2 (2–3)	1 (1–2)	0.069	2 (2–3)	2 (1–2)	0.136
Lymphadenectomy stations	6 (5–7)	5 (4–6)	0.108	6 (5–7)	5 (4.25–6)	0.245
Lymph node-positive N2 stations	0 (0–1)	0 (0–0.25)	0.297	0 (0–1)	0 (0–0)	0.145
Lymph node-positive N1 stations	0 (0–1)	0 (0–1)	0.121	0.5 (0–1)	0 (0–1)	0.178
Lymph node-positive stations	1 (0–2.5)	0 (0–1)	0.134	1 (0–2.75)	0 (0–1)	0.093
Lymphadenectomy N2	10 (7–16.5)	8.5 (5–14)	0.254	10 (7–17.75)	9 (5–16.25)	0.450
Lymphadenectomy N1	9 (5.5–10.5)	7 (4–9.5)	0.183	8.5 (5.25–10)	7.5 (4.25–11.75)	0.902
Lymphadenectomy	19 (14–25.5)	16.5 (11.75–22)	0.128	18.5 (14–26.25)	17 (12.25–24.25)	0.577
Lymph node-positive N2	0 (0–1)	0 (0–0.25)	0.298	0 (0–1)	0 (0–0)	0.139
Lymph node-positive N1	0 (0–2)	0 (0–1)	0.180	0.5 (0–2)	0 (0–1)	0.311
Lymph node-positive	1 (0–4)	0 (0–2)	0.181	1 (0–4.5)	0 (0–2)	0.157

ICU, intensive care unit.

^a^ A complex operation is defined as a surgical procedure that involves vasculoplasty or bronchoplasty.

Table 3 presents the pathological results. The pure/combined SCLC ratios between the two groups before and after the PSM were identical. All the anterior surgeries achieved complete resection, with three cases (5.1%) of microscopic residual disease in the Conventional group. The proportions of pathological stages are statistically similar between the two groups before and after PSM. However, there is a numerical difference favoring an earlier-stage disease in the Conventional group (46% vs. 35%).

**Table 3:** Pathological results before and after the propensity score matching.

Variables	Unmatched			Matched		
	HilumDirect uVATS (29)	Conventional uVATS (58)	*p*-value	HilumDirect uVATS (28)	Conventional uVATS (28)	*p*-value
Pure or Combined SCLC			1.000			0.763
Pure	22	44		21	20	
Combined	7	14		7	8	
Combined types			0.622			0.751
Pure	22	44		21	20	
Squamous cell carcinoma	0	3		0	2	
Adenocarcinoma	3	3		3	2	
Others	4	8		4	4	
SCLC cells percent	100 (99.5–100)	100 (97.5–100)	0.914	100 (99.25–100)	100 (47.5–100)	0.592
Incidental resection			0.757			0.589
No	11	24		11	13	
Yes	18	34		17	15	
Tumor location			0.447			0.101
Central	14	33		14	20	
Peripheral	15	25		14	8	
Tumor diameter	3.5 (1.9–4.5)	2.5 (1.68–4)	0.210	3.25 (1.85–4.38)	4 (2.5–5)	0.416
T stage			0.439			0.778
T1	14	34		14	11	
T2	8	16		8	11	
T3	6	5		5	4	
T4	1	3		1	2	
Elastic layer invasion			0.251			0.329
No	21	49		20	24	
Yes	8	9		8	4	
Carcinothrombosis			0.180			0.789
No	13	35		12	14	
Yes	16	23		16	14	
Nerve invasion			0.061			0.528
No	21	52		20	23	
Yes	8	6		8	5	
Resection margin			0.548			1.000
Clean	29	55		28	27	
Not Clean	0	3		0	1	
Pathological stage			0.522			0.392
I	10	26		10	13	
II	7	15		6	8	
III	12	17		12	7	
Ki67 index	70 (65–90)	80 (57.5–86.25)	0.564	70 (62.5–90)	75 (60–90)	0.789

Ki67 index, antigen Kiel 67 index.

### Survival outcomes

**Figure 3:**
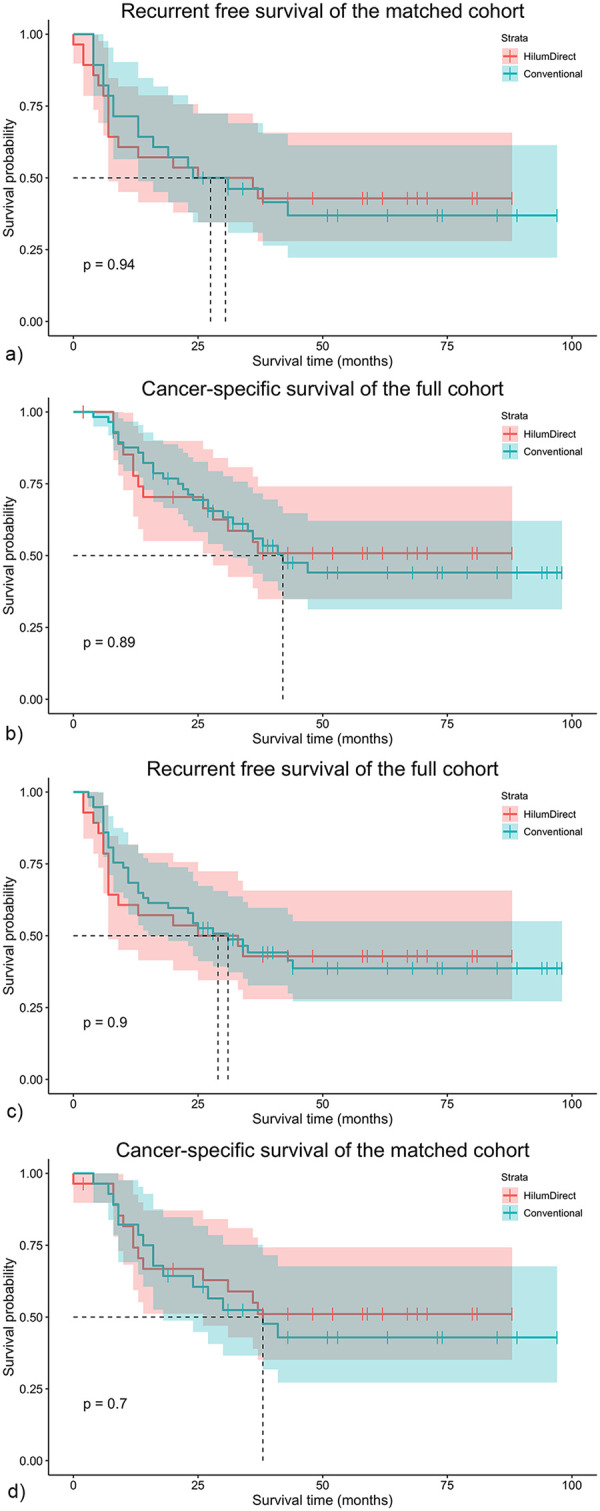
The survival results of the full cohort. The survival outcomes of the entire cohort are presented here. **(a)** Shows the CSS of the full cohort; **(b)** displays the RFS of the full cohort; **(c)** illustrates the CSS of the propensity score-matched cohort; and **(d)** depicts the RFS of the propensity score-matched cohort. CSS refers to cancer-specific survival, and RFS refers to recurrence-free survival. Anterior indicates the HilumDirect uVATS group, while Conventional denotes the Conventional uVATS group.

The median follow-up time was 32 months (2–98 months); forty cancer-specific deaths and 49 disease recurrences occurred during the time of follow-up. For the entire cohort, the median CSS time was 42 months, and the median RFS time was 32 months. The 5-year CSS rate was 47.06% (95% conf. int. 35.03%–58.19%), while the 5-year RFS rate was 40.28% (95% conf. int. 29.38%–50.91%). Before PSM, there were numerical but non-statistical differences in 5-year CSS (50.82% vs. 44.09%, *p* = 0.890) and 5-year RFS (42.86% vs. 38.46%, *p* = 0.904), with HilumDirect uVATS showing an advantage over conventional uVATS (Figures 3a,b). These numerical differences persist after PSM in the 5-year CSS (52.94% vs. 42.90%, *p* = 0.695) and 5-year RFS (44.44% vs. 36.92%, *p* = 0.940) (Figures 3c,d).

**Figure 4:**
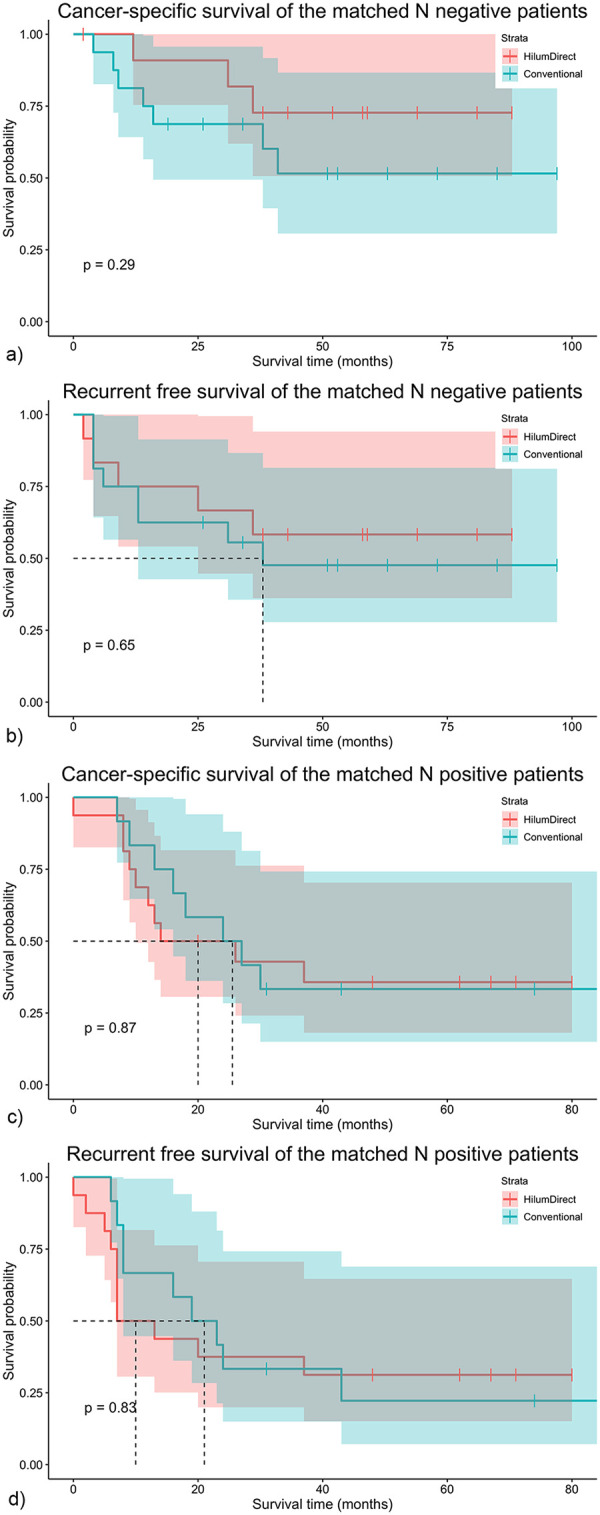
The survival results of the propensity score-matched lymph node-negative and lymph node-positive subgroups. The survival outcomes of the propensity score-matched lymph node-negative and node-positive subgroups are shown here. In **(a)**, the cancer-specific survival (CSS) of the matched lymph node-negative cohort is displayed; **(b)** illustrates the recurrence-free survival (RFS) of the matched lymph node-negative cohort; **(c)** presents the CSS of the matched lymph node-positive cohort; and **(d)** depicts the RFS of the matched lymph node-positive cohort. N negative refers to lymph node negative, and N positive indicates lymph node positive. CSS stands for cancer-specific survival, while RFS means recurrence-free survival. HilumDirect denotes the HilumDirect uVATS group, whereas Conventional refers to the Conventional uVATS group.

In the subgroup analysis, patients were stratified based on lymph node metastasis status. In the lymph node-negative stratum, the 5-year CSS rates for HilumDirect uVATS and conventional uVATS were 66.70% vs. 50.00%, *p* = 0.544, and the 5-year RFS rates for the two groups were 53.80% vs. 45.85%, *p* = 0.850, respectively. The numerical but non-statistical difference in better 5-year CSS (72.70% vs. 51.60%, *p* = 0.287) and RFS (58.30% vs. 47.60%, *p* = 0.654) rates for HilumDirect uVATS persisted after PSM (Figures 4a,b). In the lymph node-positive strata, the 5-year CSS rates for HilumDirect uVATS and conventional uVATS were 38.10% vs. 38.30%, *p* = 0.714, and the 5-year RFS rates for the two groups were 33.30% vs. 30.56%, *p* = 0.767. There appears to be a numerical difference suggesting a possible better survival rate for HilumDirect uVATS after PSM in 5-year CSS (35.70% vs. 33.30%, *p* = 0.869) and RFS (31.20% vs. 22.22%, *p* = 0.833) rates for lymph node-positive patients (Figures 4c,d).

**Figure 5:**
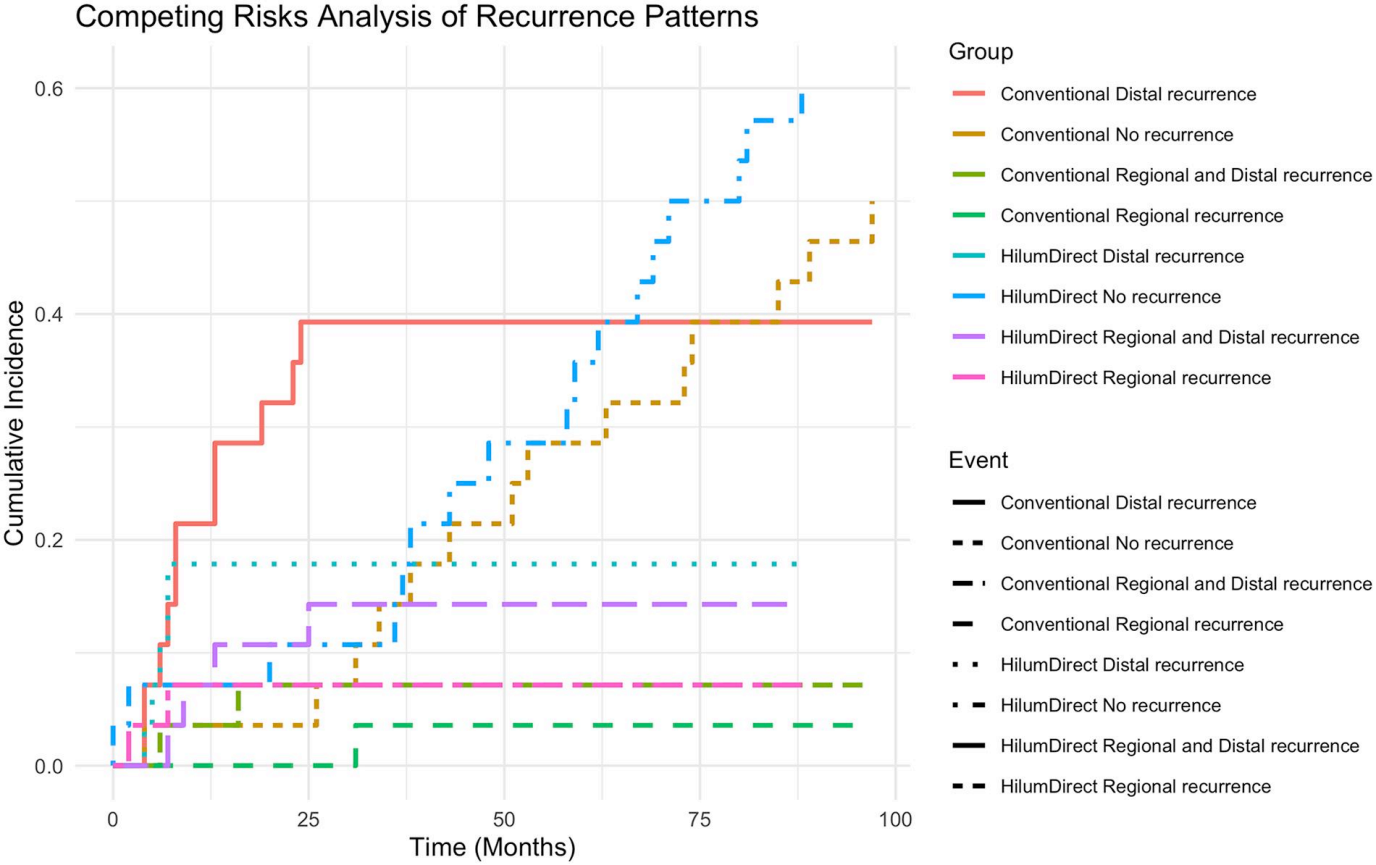
The cumulative incidence plot of competing risks analysis of recurrence patterns. This figure illustrates the recurrence patterns of the two groups: the HilumDirect uVATS group and the Conventional uVATS group. Note that there is a trend of lower risks of distal metastasis in the HilumDirect uVATS group. HilumDirect refers to the HilumDirect uVATS group, while Conventional refers to the Conventional uVATS group. No recurrence indicates death with no recurrence. Regional and distal recurrence refers to disseminated recurrence during follow-up.

A competing risks analysis was carried out in the matched groups. There is no significant difference among the competing events, namely local recurrence, distal recurrence, and mixed recurrence. However, a numerical difference suggesting a possible decrease in distal recurrence was observed. Furthermore, based on the competing risks analysis, for patients receiving Conventional uVATS compared to HilumDirect uVATS, there is also a numerical difference indicating a possible increased risk of distal metastasis (Subdistribution hazard ratio of the Conventional uVATS, sHR: 2.164, 95% CI 0.741–6.318, *p* = 0.158) (Figure 5).

Proportional hazards models were constructed to further evaluate risk factors for cancer-specific death and recurrence after treatment. For cancer-specific death, receiving neoadjuvant chemotherapy (HR 1.828, 95% conf. int. 1.122–2.979, *p* = 0.015), higher T stages (HR 3.555, 95% conf. int. 1.595–7.927, *p* = 0.002), and elevated squamous cell carcinoma antigen (HR 1.805, 95% conf. int. 1.140–2.858, *p* = 0.012) were found to be associated with a higher risk of cancer-specific death (Supplementary Table S1). For recurrence after treatment, higher T stages (HR 3.163, 95% conf. int. 1.443–6.928, *p* = 0.004) were found to be linked to a greater risk of recurrence (Supplementary Table S2).

## Discussion

Our study aimed to evaluate the safety, feasibility, and potential for improved outcomes of HilumDirect uniportal video-assisted thoracoscopic surgery (uVATS) in patients undergoing resection for small cell lung cancer (SCLC). Our findings indicate that HilumDirect uVATS is a safe and reliable procedure with comparable operative outcomes to conventional uVATS. Notably, we observed a numerical, though non-significant, difference favoring enhanced lymph node yield, cancer-specific survival (CSS), and recurrence-free survival (RFS). These findings emphasize the promise of precision-focused, minimally invasive surgical techniques in addressing the unique challenges presented by SCLC.

The central innovation of HilumDirect uVATS lies in its strategically designed incision, which sets it apart from conventional uVATS approaches. By shortening the operative distance to the lung hilum, main bronchus, and upper mediastinum, the incision enhances precision in tumor resection and lymphadenectomy. As Figure 1 shows, HilumDirect uVATS offers a wider intercostal space and larger instrument-to-chest wall angles, thus enabling broader and farther reach while facilitating the collaboration of surgical instruments compared to conventional uVATS. The significance of thorough lymph node dissection cannot be overstated in SCLC, where nodal involvement is strongly linked to recurrence and poor survival outcomes. In a database analysis, Cao et al. demonstrated a correlation between inadequate lymphadenectomy (<4 lymph nodes per resection) and worse outcomes (27). The lymph node yield, which currently varies significantly across institutions (28), is also recognized as an indicator of resection quality. In a multicenter retrospective study, Xu et al. highlighted the importance of adequate lymphadenectomy by comparing open and VATS surgery for SCLC, suggesting that a lower N2 lymph node yield (11.8 ± 8.2 vs. 8.4 ± 5.8, *p* = 0.048) correlates with a trend toward worse OS (29). In our study, the HilumDirect group exhibited a numerical difference, suggesting a potentially higher lymph node yield. Substantial evidence from large-scale research indicates a correlation between thorough lymphadenectomy and prognostic benefit. Gajra et al. have shown that extensive lymphadenectomy is associated with improved outcomes in stage I NSCLC (30). Similar correlations have been noted in other solid tumors as well (31–33). While the traditional Halsted view suggests that improved prognoses stem from clearer staging and a more exhaustive resection of tumor cells, evidence from a large-scale multicenter randomized controlled trial, i.e., Z0030, demonstrated equivalence between mediastinal lymph node sampling and systematic and thorough lymphadenectomy in presection sampling proven node-negative early-stage NSCLC (34), supporting the Cady-Fisher perspective, i.e., thorough lymphadenectomy benefits primarily through accurate staging, as cancer is a systemic disease. However, caution must be taken when interpreting our results, as a numerical but not statistical difference is insufficient for solid causal inference. Therefore, the findings of our study should be regarded as exploratory and suggestive, rather than definitive. The mechanism behind lymphadenectomy and survival remains under debate, and whether there are mechanisms other than accurate staging (35), such as reducing regional and systemic tumor burden and immunomodulation, is beyond the scope of our study.

Another theoretically plausible mechanism contributing to the observed numerical difference, which suggests possible improved survival with HilumDirect uVATS, may lie in the thermal effects of the cautery used during resection. The application of energy-based instruments could secure the resection margin by destroying residual microscopic tumor cells near the resection edge. This thermal effect was demonstrated in an *ex vivo* laryngeal model by Mannelli et al. (36). George et al. also suggested that coagulation diathermy could induce varying degrees of cellular damage compared to non-energetic instruments (37). This “sterilization effect” of cautery, while speculative, combined with the inherently broader effective resection range compared to non-energetic instruments, could help reduce local recurrence and complement systemic therapy. While our study was not designed to investigate underlying mechanisms, the observed numerical trends suggest hypotheses for future research. Future studies should aim to directly investigate the potential impact of cautery-based mechanisms on oncological outcomes in the surgical treatment of SCLC.

SCLC is known for its high metastatic potential and sensitivity to tumor cell dissemination during surgery. Conventional techniques, which often require significant retraction to ensure exposure due to the distance between the incision and the lung hilum, may inadvertently lead to excessive tumor manipulation, increasing the risk of circulating tumor cells (CTCs) entering the bloodstream or lymphatic system (15). Furthermore, certain orientations for exposure and division of the lung hilum may become inaccessible in cases of bulky disease. Single-directional VATS surgery developed by Liu et al. could address this issue by sequentially dissecting hilar structures using a triangle port design (38). However, this technique requires at least three ports, which may increase surgical trauma. The HilumDirect uVATS incision minimizes the need for extensive tissue retraction and manipulation by allowing an omnidirectional approach through a single port, as illustrated in the method section, potentially contributing to better control of SCLC. However, whether this theoretical advantage could translate into survival benefits still needs further investigation, as our study did not indicate a statistically significant superiority of the HilumDirect uVATS approach.

The assistant surgeon's training is crucial for adopting VATS surgery, as D'Amico et al. suggested (39). Kranenburg et al. systematically recorded and analyzed the ergonomic issues encountered during three-port VATS surgery (40), which were also present during the era of uVATS. The primary problems of VATS surgery generally stem from the discordance between the operative axis and the surgeon. As Bertolaccini et al. illustrated, transforming the rhomboid field from outside the thoracic cavity in multiport VATS to inside in uVATS enhanced the ergonomic experience for the surgeon (41). From a geometric perspective, the axis of HilumDirect uVATS, from the incision to the operative field, is more perpendicular to the chest wall than that of conventional uVATS, facilitating more comfortable positioning for both the surgeon and the camera-holding assistant alongside the axis. This design not only improves procedural efficiency but also creates a reproducible technique that can be easily taught to trainees.

Before interpreting the results, it is important to mention a significant statistical fact. Given the relatively small sample size (*n* = 87 total, *n* = 56 after matching), the study is at risk of a type II error, which may limit the detection of statistically significant differences. Future larger-scale studies are necessary to validate these findings.

As a cornerstone of modern SCLC management, chemoradiotherapy plays a crucial role in controlling tumor growth and enhancing survival. Evidence strongly suggests that treatment-naïve patients, particularly those with stage II disease, experience worse prognoses (42). However, Cox regression did not detect significant protective effects of neoadjuvant chemotherapy, adjuvant chemotherapy, or radiotherapy on CSS and RFS in either the whole cohort or the PSM cohort. This finding may be influenced by selection bias (43) and immortal time bias (44). Nonetheless, PSM effectively balanced neoadjuvant therapy proportions between groups, mitigating this concern to some extent. Given the impracticality of true randomization due to ethical constraints, future research may explore alternative methodologies, such as animal models and large-scale real-world database analysis, to better elucidate the true impact of multimodal therapy on surgical outcomes.

Due to inherent database limitations, detailed comorbidity records were unavailable for analysis. However, previous studies, such as those by Kearney et al. (45), have demonstrated that pulmonary reserve—measured by predicted postoperative FEV1—correlates strongly with the risk of postoperative complications. Moreover, ECOG performance status, a widely validated predictor of survival in lung cancer patients (46), was balanced between groups after matching, providing some assurance that baseline functional status was comparable. Nonetheless, pulmonary function and ECOG score are not perfect substitutes for comorbidity burden. The absence of detailed comorbidity data introduces a potential source of residual bias, which must be considered when interpreting our findings.

Surgical expertise is a critical determinant of operative quality, especially when evaluating an innovative surgical approach. While surgical proficiency varies among individuals (47), it is well established that operator experience influences surgical outcomes, particularly for complex resections. In this study, all HilumDirect uVATS cases were conducted by a single surgeon who pioneered this technique. However, technical innovation does not inherently translate into superior outcomes, as shown by the lack of significant differences in operative time, intraoperative blood loss, and other perioperative indicators. Despite these findings, the successful execution of radical resection via HilumDirect uVATS requires technical expertise and specialized training. Therefore, a measured and cautious approach is warranted when generalizing these results to broader clinical practice.

Ultimately, while our study focused exclusively on small-cell lung cancer, this disease context requires further elaboration. The biological aggressiveness and high recurrence rate of SCLC make it an especially rigorous testbed for evaluating the effectiveness of surgical techniques. In contrast, NSCLC, with its relatively slower progression and longer disease-free intervals, may obscure subtle yet meaningful differences between surgical approaches due to generally favorable outcomes. From this perspective, HilumDirect uVATS was intentionally applied to a high-risk population, grounded in the rationale that any oncological advantage observed in SCLC, even if modest, may support its efficacy and warrant broader investigation in more indolent thoracic malignancies.

This study has several limitations that warrant consideration. First, its retrospective design, conducted at a single center with a small sample size, limits the generalizability of our findings and increases the risk of a type II error. Although propensity score matching was employed, residual confounders may still exist. Specifically, the fact that all HilumDirect procedures were performed by a single surgeon who pioneered the technique introduces a potential performance bias. Furthermore, unmeasured confounders, such as detailed comorbidity data, which were unavailable in our database, could have influenced outcomes, although we attempted to mitigate this by matching on ECOG status and pulmonary function. Additionally, variations in surgeon expertise could introduce bias, and the relatively short follow-up period for some patients may underestimate long-term survival and recurrence outcomes. Perioperative factors such as pain scores and quality-of-life measures were not recorded, which might have provided deeper insights into the benefits of HilumDirect uVATS. Moreover, while numerical differences toward improved lymph node yield and survival were observed, they did not reach statistical significance, underscoring the need for larger, multicenter studies. Lastly, the underlying mechanisms contributing to these differences, such as reduced tumor manipulation, immune modulation, and thermal ablative effect, remain speculative and require further investigation.

## Conclusion

Our study demonstrates that HilumDirect uVATS is a safe and feasible surgical technique for the treatment of small cell lung cancer (SCLC). It offers comparable operative outcomes and the potential for improved results compared to conventional uVATS. The innovative incision design of HilumDirect uVATS enhances surgical precision, reduces tissue manipulation, and facilitates a more comprehensive lymphadenectomy, addressing critical challenges in SCLC resection. While numerical differences suggested possible improvements in cancer-specific survival and recurrence-free survival, particularly among node-positive patients, these findings did not reach statistical significance, highlighting the need for further validation in larger, prospective, multi-center studies.

## Data Availability

The raw data supporting the conclusions of this article will be made available by the authors, without undue reservation.
