# Supplementary material for: Safety and efficacy of the HilumDirect uVATS for small cell lung cancer: a retrospective study
Source: Front Surg. 2025 Dec 18;12:1666087. doi: 10.3389/fsurg.2025.1666087 (PMC12756433; doi:10.3389/fsurg.2025.1666087)
Supplement: Supplementary file 1 [file Datasheet1.docx]

# Lobe-Specific and Lymph Node Station-Specific Operative Approach in HilumDirect uVATS

This document describes the method of HilumDirect uVATS for lung malignancy resection. A modularized method of depiction was used. The resection of individual lobes and mediastinal lymph nodes was presented in sections.

The quintessential attribute of the HilumDirect uVATS is the allowance toward a more comprehensive usage of instruments for traction and pressing, which is unavailable in the conventional uVATS setting. In other words, our procedure enables multi-instrumental coordination, a feat previously only achievable with wide incisions in a minimally invasive setting.

## 1. Right Upper Lobectomy

### 1.1 Exposure of the right lung Hilum and Bronchial Division

1. After confirming pleural integrity, mobilize the azygos vein and its arch for exposure of the right upper lobe hilum.
2. Expose the posterior hilum by gently retracting the lung tissue, ensuring adequate visualization of the vascular and bronchial structures.
3. The right upper lobar bronchus is transected at the division level, preferably using diathermy to minimize the stump size. The bronchial stump is then closed with a continuous suture.
4. Dissect the anterior mediastinal pleura to visualize the truncus anterior artery and upper pulmonary vein fully.

### 1.2 Vascular and Fissure Division

1. The truncus anterior branch of the pulmonary artery is identified, controlled, and divided with transfixion or ligation, or vascular staplers. The distal upper lobar artery branches are then dissected and divided similarly.
2. The right upper pulmonary vein is then dissected and divided using transfixion, ligation, or staplers. The majority of the right upper lobar 12R station lymph nodes should be freed along with the lobar parenchyma.
3. The fissures are divided to release the upper lobe fully. Note that low-tidal-volume bilateral ventilation can be applied to fully delineate the fissures. The right 11R station lymph nodes can be freed along with the lobar parenchyma.
4. The order of division could be altered to meet individualized anatomical and oncological conditions.

## 2. Right Middle Lobectomy

### 2.1 Exposure and Mobilization

1. The hilar pleura is dissected to expose the middle pulmonary vein. The next step depends on the condition of the oblique and horizontal fissures.
   - If the fissures are well-developed, the pulmonary artery and exposed pulmonary vein can be divided using ligation, transfixion, or staplers, ensuring hemostatic control.
   - If the fissures are underdeveloped, the middle lobar bronchus can be exposed and adequately freed through the lower part of the anterior hilum, between the two pulmonary veins. To sufficiently mobilize the middle lobe before addressing the fissures requires diathermal cutting of the middle lobar bronchus, followed by continuous suture of the stump.

### 2.2 Hilar Structures Division and Removal of the Lobe

1. Depending on the condition of the fissures, the division order of the pulmonary artery, vein, and bronchus may vary, while the principle of management of those structures remains the same.
   - For the middle lobe with well-developed fissures, the dissection and division of the bronchus could be conducted after the management of the vessels.
   - For the middle lobe with underdeveloped fissures, dissecting and dividing the bronchus first can effectively untether the middle lobe, easing the subsequent steps. The low-tidal-volume bilateral ventilation could be used to visualize the fissure if needed.
2. After dissecting and division of the middle lobar hilar structures, the pulmonary fissures are then divided by diathermal cutting or staplers.
3. The lymph nodes adjacent to the middle lobar bronchus (12R station), the intermediate bronchus (11R station), and the lower lobar bronchus (12R station) can be dissected and removed along with the middle lobe in an en-bloc fashion.

## 3. Right Lower Lobectomy

### 3.1 Division of the Inferior Pulmonary Ligament

1. Mobilize the inferior pulmonary ligament to improve exposure; during this process, the 9R station and 8 station lymph nodes are harvested.
2. Dissect around the inferior part of the right lung hilum along the pericardium to expose the inferior pulmonary vein and free the medial side of the lymph nodes adjacent to the right lower bronchus.

### 3.2 Vascular and Bronchial Division

1. The order of vascular dissection and division depends on the condition of the oblique fissure:
   - When the oblique fissure is underdeveloped, dissect and divide the pulmonary vein first, then to the bronchus, and the right lower pulmonary artery will be the last. Pay attention to the origin of the middle lobar pulmonary artery, as a distal-originated middle lobar artery branch is prone to injury during blunt dissection.
   - When the oblique fissure is well-developed, the order of dissection and division can vary depending on the location of the tumor. The methods of vascular and bronchial control and division do differ.
2. Whenever the right lower bronchus is divided, the adjacent hilar lymph node can be dissected and retrieved along with the lower lobe.

### 3.3 Division of the Fissure and Retrieval of the Lobe

1. The oblique fissure can be divided if it is underdeveloped. Diathermal cutting is more fitting if the interlobar lymph nodes (11R) need thorough dissection.
2. The lobe can be retrieved once freed, ideally along with the 10R station, the 11R station, and the 12R station, to conform to the en bloc principle.

## 4. Left Upper Lobectomy

### 4.1 Hilar Dissection and Mobilization

1. Dissect the upper part of the left lung hilum to expose the left pulmonary artery trunk, the upper hilar lymph nodes (10L), and the aortopulmonary window lymph nodes (5) can be dissected and harvested.
2. Dissect the left upper hilum to mobilize the upper pulmonary vein adequately.
3. Identify the lingular and apical branches of the pulmonary artery if the oblique fissure developed well.

### 4.2 Hilar Structure and Oblique Fissure Division

1. The division order of hilar structures depends on the development of the oblique fissure:
   - If the oblique fissure is well-developed, the upper lobar branches of the pulmonary artery can be managed with ligation, transfixion, or staplers first if needed. The dissection and division of the left upper lobar bronchus can be conducted either before or after the management of the left upper vein.
   - If there is an underdeveloped fissure, the order can be vein-bronchus-artery to ensure adequate exposure of the bronchus when dissecting and dividing. A bronchus-first approach can also be conducted if the untethering of the left upper lobe is imminent.
2. The lymph nodes adjacent to the left upper lobar bronchus can be dissected and harvested along with the left upper lobe.
3. Either before or after the dissection and division of the hilar structures of the left upper lobe, the oblique lobe can be divided by diathermal cutting or staplers.
4. The left upper lobe and its adjacent lymph nodes can now be retrieved in an en-bloc fashion.

## 5. Left Lower Lobectomy

The order and the methods used for the dissection and division of relevant structures do not differ significantly from those for the right lower lobectomy, except for the dissection and division of the left lower pulmonary vein, which requires transfixion or multiple ligation if the pericardium hinders the entry and fire of the staplers.

### 3.1 Division of the Inferior Pulmonary Ligament

1. Mobilize the inferior pulmonary ligament to improve exposure; during this process, the 9R station and 8 station lymph nodes are harvested.
2. Dissect around the inferior part of the left lung hilum along the pericardium to expose the inferior pulmonary vein and free the medial side of the lymph nodes adjacent to the left lower bronchus.

### 3.2 Vascular and Bronchial Division

1. The order of vascular dissection and division depends on the condition of the oblique fissure:
   - When the oblique fissure is underdeveloped, dissect and divide the pulmonary vein first, then to the bronchus, and the left lower pulmonary artery will be the last.
   - When the oblique fissure is well-developed, the order of dissection and division can vary depending on the location of the tumor. The methods of vascular and bronchial control and division do differ.
2. Whenever the left lower bronchus is divided, the adjacent hilar lymph node can be dissected and retrieved along with the lower lobe.

### 3.3 Division of the Fissure and Retrieval of the Lobe

1. The oblique fissure can be divided if it is underdeveloped. Diathermal cutting is more fitting if the interlobar lymph nodes (11R) need thorough dissection.
2. The lobe can be retrieved once freed, ideally along with the 10R station, the 11R station, and the 12R station, to conform to the en bloc principle.

## 6. Right Lymphadenectomy

### 6.1 Subcarinal (Station 7) and Hilar (Stations 10R, 10L) lymphadenectomy

Subcarinal (7) and hilar (10R, 10L) nodes, which reside adjacent to the median wall of the main bronchi, can be reached both anteriorly and posteriorly through the incision of HilumDirect uVATS.

For the anterior approach, a tender press on the right pulmonary artery will sufficiently expose the subcarinal region. The regional lymph nodes are then harvested in an en-bloc fashion. The left and right boundaries of the lymph nodes group would obviously be the respective main bronchi. However, caution must be taken to avoid injuring the posteriorly located esophagus, the anteriorly located right pulmonary trunk, and the aortic arch.

The mediastinal pleura behind the right main and intermediate bronchi can be opened if needed. This bi-directional approach to this area allows more efficient and complete lymphadenectomy, as it facilitates the exposure of the anterior adjacent structures.

### 6.2 Right Paratracheal (Stations 2R, 4R) lymphadenectomy

The azygos arch is optimally mobilized by dissecting its flanking mediastinal pleura. The upper hilar lymph nodes (10R), which are caudally and medially located relative to the arch, and the portion of the 4R lymph nodes, which are cranially and medially located relative to the arch, can be fully exposed at this time.

With optimal exposure, the 10R and 4R lymph nodes can be fully dissected with the traction of the azygos arch. The shorter incision-to-field distance and larger movement angle allow for complete exposure of this area, and the adjacent trachea, the carina, the main bronchi, the right pulmonary artery trunk, and the posterior wall of the superior vena cava can be visualized with ease to achieve en-bloc resection of lymph nodes.

Once the 10R and 4R lymph nodes are freed, the dissection can be moved cranially toward the 2R station area. Cautions are needed to identify the right vagus nerve and the right laryngeal recurrent nerve. HilumDirect uVATS allows for at least two instruments other than the thoracoscope, the diathermy, and the suction apparatus to apply traction and press to allow precise dissection in this area, which is usually too far to reach in conventional uVATS.

The posterior adventitia of the right brachiocephalic and the subclavicular vein, the right side of the adventitia of the brachiocephalic artery, and the anterior wall of the trachea will be fully exposed after the dissection of the 2R station lymph nodes. The 4R, 2R, and adjacent 10R lymph nodes can now be retrieved in an en-bloc fashion.

### 6.3 Other stations of lymph nodes

For the 3a lymph nodes, the mediastinal pleura adjacent to the anterior adventitia of the superior vena cava is opened first. The right internal thoracic vein is then exposed to ensure adequate exposure and prevent accidental injury. The 3a lymph nodes can then be dissected away from the pericardium till complete en-bloc resection.

## 7. Left Mediastinal Lymphadenectomy

With the HilumDirect uVATS approach, the distance from the incision to the left upper mediastinum can be significantly shortened, and the movement angle of the instrument broadened. Allowing a thorough mediastinal lymphadenectomy to be conducted in the uniportal setting.

For example, the control and division of the ligamentum arteriosum can be performed safely with multiple ligations and sharp dissection without the need for clips or staplers. A more direct approach to the upper mediastinum also safeguards the operation by providing the capacity for quickly applicable vascular control in case of incidental injury.

### 7.1 The anterior upper hilar region (Stations 5, 6, 10L)

The mediastinal pleura between the phrenic nerve and the left vagus nerve is longitudinally opened to reach optimal exposure of the aortopulmonary region.

Free the hilar lymph nodes first to visualize the distant end of the left pulmonary artery trunk. Then, cranially dissect the plane between the adventitia and the regional connective tissue containing lymph nodes. Keep the dissection plane in this space until the ligamentum arteriosum is exposed. The ligamentum arteriosum can be controlled and divided if a more thorough lymphadenectomy is warranted, provided that a patent ductus arteriosus is ruled out by preoperative imaging.

The plane of dissection now turns to that between the area of lymph nodes and the left adventitia of the aortic arch. With the dissection plane moving anteriorly, the lymph node stations 5, 6, and 10L can be dissected and retrieved eventually.

### 7.2 The posterior upper hilar region (Stations 4L and 10L)

This area of lymph nodes contains the 4L, 10L, and those adjacent to the left laryngeal recurrent nerve. The mediastinal pleura between the left upper pulmonary artery and the descending aorta is opened first to expose the lower trachea and the left upper lobar bronchus.

The vagus nerve and the left laryngeal recurrent nerve are reliable landmarks for lymphadenectomy in this area. Made available to more instruments, the left pulmonary artery can be pressed to ensure optimal exposure and prevent injury to the pulmonary artery in the process. Blunt dissection is preferred to protect the left laryngeal recurrent nerve from electrical and thermal damage. The inter-tissue plane of the area of lymph nodes and the pulmonary artery is then divided.

The plane between the aorta and the area of lymph nodes is dissected next. Pay attention to the possible artery that branches off from the aorta, as dehiscence leads to catastrophic hemorrhage. Control by ligation or transfixion can be performed with ease in the HilumDirect uVATS setting. The targeted lymph nodes are then dissected off the tracheal and bronchial walls. The stations 4L and 10L lymph nodes can be retrieved in an en-bloc fashion, finally.

### 7.3 Subcarinal (Station 7) and Hilar (Stations 10R, 10L) lymphadenectomy

This area of lymph nodes can be resected through a posterior or anterior approach. The latter requires division of the left upper pulmonary vein to gain optimal exposure of the area. Thus, it is available in the left upper lobectomy setting.

For the anterior approach. Additional instruments are used to elevate the left main bronchus and press down the pericardium. The inter-tissue plane between the area of the lymph nodes and the pericardium is dissected first. After freeing the targeted lymph nodes to some extent, the inter-tissue plane between the area of the lymph nodes and the left bronchus is dissected. The deepest inter-tissue plane between the area of lymph nodes and the right bronchus is dissected using traction and pressing instruments. The targeted lymph nodes can then be retrieved in an en-bloc fashion.

For the posterior approach, the parenchyma is gently pressed anteriorly to expose the subcarinal area. With the appropriate application of instruments for traction and pressing, the targeted lymph nodes will be resected and retrieved in a fashion similar to another area of lymph nodes.
